# Catastrophic health expenditure among Chinese adults living alone with cognitive impairment: findings from the CHARLS

**DOI:** 10.1186/s12877-022-03341-8

**Published:** 2022-08-04

**Authors:** Chenxi Li, Shuyi Jin, Xingqi Cao, Ling Han, Ning Sun, Heather Allore, Emiel O. Hoogendijk, Xin Xu, Qiushi Feng, Xiaoting Liu, Zuyun Liu

**Affiliations:** 1 Department of Big Data in Health Science School of Public Health and Center for Clinical Big Data and Analytics of the Second Affiliated Hospital, The Key Laboratory of Intelligent Preventive Medicine of Zhejiang Province, Zhejiang University School of Medicine, 866 Yuhangtang Rd, Zhejiang, 310058 Hangzhou China; 2 Department of Internal Medicine, Yale School of Medicine, New Haven, CT USA; 3 Ningbo College of Health Sciences, Ningbo, Zhejiang, China; 4 Department of Epidemiology & Data Science, Amsterdam Public Health research institute, Amsterdam UMC – location VU University medical center, Amsterdam, the Netherlands; 5 Department of Sociology, National University of Singapore, Singapore, Singapore; 6 School of Public Affairs, Zhejiang University, 866 Yuhangtang Rd, Zhejiang, 310058 Hangzhou China

**Keywords:** Catastrophic health expenditure, Living alone, Cognitive impairment, Chinese adults

## Abstract

**Background:**

The catastrophic health expenditure of older adults results in serious consequences; however, the issue of whether cognitive status and living situations contribute to such financial burdens is uncertain. Our aim was to compare the differences in catastrophic health expenditure between adults living alone with cognitive impairment and those adults living with others and with normal cognition.

**Methods:**

We identified 909 observations of participants living alone with cognitive impairment (cases) and 37,432 observations of participants living with others and with normal cognition (comparators) from the 2011/2012, 2013, 2015 and 2018 waves of the China Health and Retirement Longitudinal Study (CHARLS). We used propensity score matching (1:2) to create matched cases and comparators in a covariate-adjusted logistic regression analysis. Catastrophic health expenditure was defined as an out-of-pocket cost for health care ≥40% of a household’s capacity to pay.

**Results:**

In comparison with participants living with others and with normal cognition, those adults living alone with cognitive impairment reported a higher percentage of catastrophic health expenditure (19.5% vs. 11.8%, respectively, *P* < 0.001). When controlling for age, sex, education, marital status, residence areas, alcohol consumption, smoking status and disease counts, we found that this subpopulation had significantly higher odds of having catastrophic health expenditure (odds ratio [OR] = 1.89, 95% confidence interval [CI]: 1.40, 2.56). Additional analyses confirmed the robustness of the results.

**Conclusions:**

This study demonstrated that adults living alone with cognitive impairment in the CHARLS experienced a high burden of catastrophic health expenditure. Health care policies on social health insurance and medical assistance should consider these vulnerable adults.

**Supplementary Information:**

The online version contains supplementary material available at 10.1186/s12877-022-03341-8.

## Introduction

Mild cognitive impairment is highly prevalent in the older Chinese population, and it has been estimated to affect 15.2–15.9% of Chinese adults aged 60 years and greater, which corresponds to 38.0–39.6 million people [1]. Studies in European countries and the United States have suggested that a large proportion of adults with cognitive impairment live alone (between 28 and 34%) [2, 3]. The health care burden of these vulnerable adults has aroused public concern [4]. Recently, the University of California San Francisco has been establishing the Living Alone with Cognitive Impairment Project, with the goal of enhancing the well-being of adults living alone with cognitive impairment. In developing Asian countries (including China), little is known about these vulnerable adults because traditional filial piety cultural norms encourage cohabitation with children and the administration of care from family members [5]. It is likely that adults with cognitive impairment living alone are socially disadvantaged. However, there are limited population-level studies on the health care burdens of these vulnerable adults in Asian countries, thus resulting in an obstacle for policy-makers for developing appropriate intervention programs.

Catastrophic health expenditure represents an important indicator of the excessive financial burden due to out-of-pocket (OOP) health care costs, which may place households under a situation of unanticipated financial catastrophe or impoverishment [6, 7]. When OOP payments for health care equal or exceed 40% of the household’s capacity to pay, households may face catastrophic health expenditure. In response to this problem, governments throughout the world have made significant efforts to develop a universal medical insurance system [8]. Despite the considerable efforts of governments in resolving the issue of medical insurance coverage, it has fallen short of providing financial protection against the medical expenditure burden. Therefore, we speculate that the medical insurance system should focus on the vulnerable subpopulation to improve the overall efficiency. Previous studies have indicated that older adults living alone may be at higher odds of incurring catastrophic health expenditure due to lower household worth (i.e., the lower socioeconomic status), a higher prevalence of physical multimorbidity and limited access to health services relative to their normal counterparts [9, 10]. However, living alone can only explain a small fraction of high health expenditure in adults, and some adults living alone even have better health statuses with lower health expenditure compared to those adults living with family members [11]. There are other factors associated with health expenditure and the management of finances, such as cognitive impairment [7]. To the best of our knowledge, no studies have evaluated the odds of incurring catastrophic health expenditure among adults living alone with cognitive impairment. Previous comparative studies of living alone or cognitive impairment have not appropriately addressed data imbalance and confounding factors, and most of the studies were conducted in developed countries. Modern analytic methods in the field of causal inference, such as propensity score matching, allow for a balance of confounding factors. Studies have shown that propensity scoring methods for observational studies may lead to unbiased estimates of treatment effects [12, 13], and can address some limitations of standard multivariable regression models [14, 15]. Therefore, in the present study, our aim was to investigate whether adults living alone with cognitive impairment had higher odds of incurring catastrophic health expenditure in comparison with those adults living with others and with normal cognition by applying propensity score matching to address imbalances and confounding factors. We used data from an ongoing nationally representative survey of adults ≥45 years old from the China Health and Retirement Longitudinal Study (CHARLS).

## Methods

### Study observations

The CHARLS was a nationally representative longitudinal survey targeting Chinese community-dwelling individuals aged 45 years and older along with their spouses and used multistage stratified probability-proportionate-to-size sampling to cover 28 provinces, 150 countries/districts and 450 villages/urban communities across China [16]. The CHARLS collected information on various demographic characteristics, physical function, chronic disease, family structure, work, socioeconomic status, retirement and pension, health care and insurance, income, and consumption. Additionally, we used data from four waves (i.e., 2011/2012, 2013, 2015 and 2018) of the CHARLS. The details of the CHARLS are provided in a previous study [16].

**Fig. 1:**
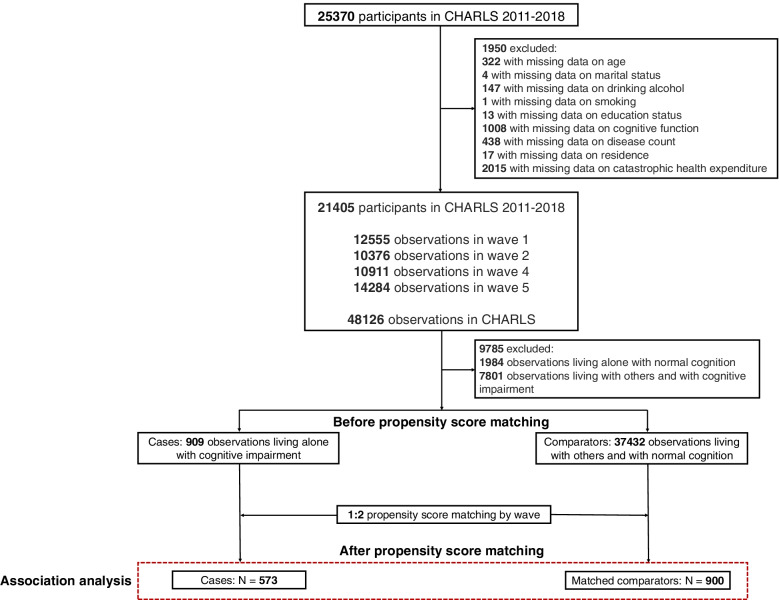
Detailed steps for the selection of the study observations

Participants were involved in one or more waves of the CHARLS, and their cohabitation and cognitive function may vary across waves. Therefore, the study unit was defined as an observation rather than a participant. As shown in Fig. 1, we first excluded 1950 participants due to missing covariate data from a total of 25,370 participants in any of the 4 waves of the CHARLS. Among the 48,126 observations from the remaining 21,405 participants, we excluded 1984 observations of participants living alone with normal cognition and 7801 observations of participants living with others and with cognitive impairment. Thus, we identified 909 observations of participants living alone with cognitive impairment (cases) and 37,432 observations of participants living with others and with normal cognition (comparators).

### Measurement of cognitive impairment

Based on similar concepts in the Health and Retirement Study (HRS), the CHARLS designed a questionnaire evaluating two cognition measures, including episodic memory and executive function. Episodic memory was measured by the immediate and delayed recall of words. Ten unrelated Chinese words were read to each participant, and his or her memory ability was evaluated by adding up the number of correct words that were immediately recalled (immediate word recall scores) and recalled 4 minutes later (delayed word recall scores). We calculated episodic memory as the average scores of immediate and delayed word recall scores [17], with a range of 0 to 10 [17, 18]. Episodic memory is a necessary component of reasoning in many dimensions. Executive function was based on the components of the mental status questions of the Telephone Interview of Cognitive Status (TICS) and figure drawing [19]. TICS is a reliable and valid method as the Mini-Mental State Examination (MMSE), and is used to screen cognitively impaired elderly individuals [20]. Components in TICS included today’s date (month, day, year and seasons), the day of the week and the serially subtraction of 7 from 100 (5 times). In figure drawing, participants were asked to redraw a picture from one painting. We calculated executive function as the total score from TICS and figure drawing, ranging from 0 to 11. The cognitive function score was the sum of episodic memory and executive function, with a higher score indicating better cognitive function (range: 0–21) [21]. As has been reported in the literature [22], we defined the observation as having cognitive impairment if the summary score was less than 6; otherwise, the observation was defined as having normal cognition.

### Measurement of living alone

The CHARLS used the household roster file to collect the number of residents living in a household, including all of the reported members living in the household, except for the household respondents and their spouse (as identified by the household respondents). Living alone was defined as the number of residents living in the household being one [23], and living with others was defined as the number of residents living in the household being greater than one.

### Measurement of catastrophic health expenditure

Self-reported information on money (including the fact that participants paid OOP for their last month’s outpatient visits and last year’s inpatient visits) was collected in the CHARLS. The spouses of all of the participants were collected for the same information. Each participant’s annual OOP cost on outpatient care was calculated as the result of multiplying the last month’s cost by 12 [6].

A household’s capacity to pay was defined as the total cost of the household’s consumption minus the food-based household cost [6]. A household’s OOP cost on health care was defined as the sum of the annual OOP cost on inpatient and outpatient health care of both the participant and his or her spouse [6]. A household was defined as incurring catastrophic health expenditure if the OOP cost on health care was ≥40% of a household’s capacity to pay [24, 25]. In particular, for those participants who did not have spouses living together in a household, we considered their spouses’ annual OOP costs on health care to be 0. Furthermore, a binary variable was defined to indicate whether there was catastrophic health expenditure in the participant’s household, which has been widely used in previous studies based on the CHARLS [6, 26, 27].

### Covariates

We considered a series of covariates that may be associated with living alone, cognitive impairment [28–34] or catastrophic health expenditure [35]. These covariates included age, sex (male vs. female), residence areas (rural vs. others [“city/town”, “combination zone between urban and rural areas” and “special area”]), marital status (currently married vs. others including “separated”, “divorced”, “widowed” and “never married”), education (no schooling vs. primary school or more), alcohol consumption (non-drinker vs. drinker; we defined “alcohol consumption” via the question “Did you drink any alcoholic beverage, such as beer, wine, or liquor in the past year? How often?”. An observation was defined as a non-drinker if his or her answer was “None of these” and as a drinker if his or her answer was “Drink but less than once a month” or “Drink more than once a month”), smoking status (non-smoker, ever smoker and current smoker) and disease counts. Disease counts were calculated based on the number of self-reported diseases diagnosed by doctors, including hypertension, cancer, diabetes, lung disease, stroke, heart disease, arthritis, kidney disease, asthma and digestive disease.

### Statistical analyses

In the descriptive analysis, the mean ± SD was used for the continuous variables, and numbers and percentages were used for the categorical variables, unless otherwise specified.

To address data imbalance and confounding factors between cases and comparators, propensity score matching was used. A propensity score is the conditional probability of an exposure for a set of covariates [36], which was estimated by using a multivariable logistic regression model [37]. The dependent variable was living alone with cognitive impairment, and the independent variables were age, sex, education, marital status, residence areas, smoking status, alcohol consumption and disease counts. A 1:2 matching protocol was used for matching without replacement (the greedy-matching algorithm), and the caliper width was equal to 0.2 of the standard deviation of the logit of the propensity score. We estimated the standardized mean differences (SMDs) for all of the covariates before and after matching to assess prematch imbalance and postmatch balance. For a given covariate, a SMD of < 10.0% represented a relatively small imbalance [38].

To investigate whether adults living alone with cognitive impairment had a higher percentage of catastrophic health expenditure than those adults living with others and with normal cognition, we compared the cases and the matched comparators. We first compared distributions of outcomes (i.e., catastrophic health expenditure) of observations between the cases and the matched comparators by using Mann–Whitney U tests for the continuous variables and the chi-square test for the categorical variables. Subsequently, generalized estimating equation models [39] were used to estimate the odds ratio (OR) and corresponding 95% CIs of catastrophic health expenditure for the cases relative to the matched comparators. To account for the correlation between observations from the same participant, we used the logit link function and autoregressive correlation matrix. We considered two models. Specifically, Model 1 adjusted for age and sex, and Model 2 additionally adjusted for education, marital status, residence areas, alcohol consumption, smoking status and disease counts.

We performed three additional analyses to test the robustness of our findings. First, we changed the cut-off value for defining cognitive impairment to a summary score that was at least one standard deviation (SD) below age-appropriate norms and then re-examined the association with the same models. Subsequently, we added ADL status as a covariate and re-examined the association with the same model. Finally, we reperformed the analysis without adjusting for marital status.

R version 4.1 and SAS version 9.4 (SAS Institute, Cary, NC) were used to perform all of the statistical analyses. Statistical significance was defined as a *P* value less than 0.05 (two-tailed).

## Results

Table 1 shows the characteristics of the observations in the cases (i.e., living alone with cognitive impairment), the comparators and the matched comparators (i.e., living with others and with normal cognition). Before propensity score matching, the cases reported a higher percentage of catastrophic health expenditure relative to the comparators (19.7% vs. 18.1%, respectively, *P* = 0.229), but the difference was nonsignificant. After propensity score matching, the cases reported a significantly higher percentage of catastrophic health expenditure relative to the matched comparators (19.5% vs. 11.8%, respectively, *P* < 0.001).

**Table 1:** Characteristics of observations in the cases (i.e., living alone with cognitive impairment) and the comparators (original and matched, i.e., living with others and with normal cognition) in CHARLS 2011–2018

	Before Propensity Score Matching				After Propensity Score Matching			
	Case (***n*** = 909)	Original comparators (***n*** = 37,432)	***P*** value	SMD	Case (***n*** = 573)	Matched comparators (***n*** = 900)	***P*** value	SMD
**Covariates**								
Wave			< 0.001	0.442			0.942	0.033
1	189 (20.8)	9932 (26.5)			166 (29.0)	259 (28.8)		
2	128 (14.1)	8503 (22.7)			102 (17.8)	151 (16.8)		
4	159 (17.5)	8857 (23.7)			106 (18.5)	175 (19.4)		
5	433 (47.6)	10,140 (27.1)			199 (34.7)	315 (35.0)		
Age, years	71.9 ± 9.5	58.1 ± 8.6	< 0.001	1.524	69.5 ± 9.6	67.8 ± 9.5	< 0.001	0.180
Middle-aged adults (45–59, years)	93 (10.2)	22,004 (58.8)	< 0.001	1.188	85 (14.8)	182 (20.2)	0.011	0.142
Older adults (≥ 60, years)	816 (89.8)	15,428 (41.2)			488 (85.2)	718 (79.8)		
Sex			< 0.001	0.445			0.867	0.012
Female	623 (68.5)	17,632 (47.1)			370 (64.6)	576 (64.0)		
Male	286 (31.5)	19,800 (52.9)			203 (35.4)	324 (36.0)		
Marital status			< 0.001	4.177			0.264	0.066
Currently married	39 (4.3)	35,365 (94.5)			39 (6.8)	77 (8.6)		
Others	870 (95.7)	2067 (5.5)			534 (93.2)	823 (91.4)		
Residence areas			< 0.001	0.469			0.103	0.091
Rural	744 (81.8)	22,932 (61.3)			433 (75.6)	644 (71.6)		
Others	165 (18.2)	14,500 (38.7)			140 (24.4)	256 (28.4)		
Alcohol consumption			< 0.001	0.253			0.737	0.021
Non-drinker	615 (67.7)	20,751 (55.4)			368 (64.2)	587 (65.2)		
Drinker	294 (32.3)	16,681 (44.6)			205 (35.8)	313 (34.8)		
Smoking status			< 0.001	0.182			0.836	0.032
Non-smoker	580 (63.8)	20,560 (54.9)			361 (63.0)	562 (62.4)		
Ever smoker	133 (14.6)	6584 (17.6)			79 (13.8)	118 (13.1)		
Current smoker	196 (21.6)	10,288 (27.5)			133 (23.2)	220 (24.4)		
Education			< 0.001	1.378			0.003	0.162
No schooling	640 (70.4)	5366 (14.3)			320 (55.8)	430 (47.8)		
Primary school or more	269 (29.6)	32,066 (85.7)			253 (44.2)	470 (52.2)		
Disease counts	1.8 ± 1.5	1.5 ± 1.4	< 0.001	0.206	1.7 ± 1.5	1.6 ± 1.5	0.262	0.067
0	195 (21.5)	10,313 (27.6)	< 0.001	0.213	134 (23.4)	205 (22.8)	0.077	0.139
1	269 (29.6)	11,248 (30.0)			171 (29.8)	299 (33.2)		
2	181 (19.9)	8129 (21.7)			108 (18.8)	194 (21.6)		
≥ 3	264 (29.0)	7742 (20.7)			160 (27.9)	202 (22.4)		
**Outcomes**								
Catastrophic health expenditure			0.229	**─**			< 0.001	**─**
Yes	179 (19.7)	6768 (18.1)			112 (19.5)	106 (11.8)		
No	730 (80.3)	30,664 (81.9)			461 (80.5)	794 (88.2)		

*CHARLS* China Health and Retirement Longitudinal Study; *SMD* standardized mean difference

“Others” for marital status included “separated”, “divorced”, “widowed” and “never married”

“Others” for residence areas included “city/town”, “combination zone between urban and rural areas”, and “special area”

Mann-Whitney U test was used for two continuous variables (age and disease counts). Chi-square test was used for the rest of the variables. All the tests were used to present the differences between the cases and the matched comparators with P value showing the significance

Data is shown as mean ± standard deviation or numbers (percentages)

Percentages may not add up to 100 because of rounding

As shown in Table 2, after adjustments for a series of covariates (i.e., age, sex, education, marital status, residence areas, alcohol consumption, smoking status and disease counts), compared to the matched comparators, the statistical models suggested that the cases had significantly higher odds of catastrophic health expenditure (OR = 1.89, 95% CI: 1.40, 2.56).

**Table 2:** Catastrophic health expenditure in the cases (i.e., living alone with cognitive impairment) and the matched comparators (i.e., living with others and with normal cognition) in CHARLS 2011–2018

	Model 1 OR (95% CI)	***P*** value	Model 2 OR (95% CI)	***P*** value
**Catastrophic health expenditure**	1.91 (1.42, 2.57)	< 0.001	1.89 (1.40, 2.56)	< 0.001

*OR* odds ratio; *CI* confidence interval

Generalized estimating equation models were used for catastrophic health expenditure. Model 1 adjusted for age and sex. Model 2 additionally adjusted for marital status, residence areas, alcohol consumption, smoking status, education, and disease counts based on Model 1

In the additional analyses, we found that the results were consistent with the main findings when we defined cognitive impairment by using another cut-off of at least one SD below age-appropriate norms, added ADL status as a covariate and did not adjust for marital status (Table S1-S3).

## Discussion

The current study focused on middle-aged and older adults living alone, which is a subpopulation that is growing rapidly in many countries, including China. These vulnerable adults require increased focus on policy [4], especially if they are experiencing cognitive impairment. To our knowledge, this was the first study focusing on the health expenditure of adults living alone with cognitive impairment. This study was based on a large nationally representative longitudinal cohort of noninstitutionalized adults aged ≥45 years in China. After propensity score matching, we observed that adults living alone with cognitive impairment in the CHARLS had significantly higher odds of incurring catastrophic health expenditure than those adults living with others and with normal cognition. The findings demonstrated the increased burden of health expenditure in these vulnerable adults.

Many reasons, such as poor economic status, decreased family size, massive population migration and longer lifespans of women [40], may lead to living alone with cognitive impairment, especially in developing countries, including China, where pension service quality is still poor [41]. This was manifested as unique characteristics of this vulnerable subpopulation that were observed in our study (e.g., low education level, Table 1). The substantial differences in characteristics between this vulnerable subpopulation and the general populations result in difficulties when performing traditional standard multivariable regression analyses. Our results underscore the increased burden of health expenditure in adults living alone with cognitive impairment, even when factors were well-controlled for potential confounding effects from these unique characteristics.

The underlying mechanisms of the observed relationship between living alone and cognitive impairment and catastrophic health expenditure are not well understood. Previous studies have demonstrated consistent findings that physical multimorbidity was positively associated with an increased risk of catastrophic health expenditure [9, 42], and previous studies have found that the number of concurrent conditions is associated with an increased risk of developing cognitive impairment [43, 44]. Interestingly, in contrast to our expectations, we did not observe a significant difference in disease counts between the cases and the matched comparators. It is possible that physical multimorbidity is underreported among these vulnerable adults, due to the fact that the CHARLS collected self-reported diseases. This scenario is highly likely when considering that over half of the cases had no schooling. Physical disability may be another reason that explains these findings, as living alone with cognitive impairment was positively associated with physical disability in previous studies [23]. It is obvious that physical disability may stimulate increased health expenditure and OOP costs, which tend to place a heavy financial burden and catastrophic health expenditure on these vulnerable adults. Therefore, future studies could shift focus on accessibility to primary health care to physical multimorbidity and physical disability prevention, management, as well as treatment, to improve the effectiveness of health care policies in mitigating these inequalities, with special consideration given to these vulnerable adults.

In addition, adults living alone with cognitive impairment may represent those individuals at low socioeconomic levels in the population and those individuals who were not covered by Urban Resident Basic Medical Insurance (URBMI). The URBMI, which is one of the basic social insurance schemes in China, was initiated in 2007 and formally launched in 2009 to cover urban residents (except for urban employees), including children, students, elderly people without previous employment and unemployed people. A large proportion (approximately 2/3 individuals) of the annual premium is contributed by the government, whereas individuals pay for a small proportion (1/3 individuals of the total premium). The URBMI funds are pooled at the prefectural/municipal level and managed by the previous Ministry of Human Resource and Social Security [45]. From the time of 2016, URBMI and the New Rural Cooperative Medical System (NRCM) have been integrated into the Basic Insurance Scheme for Rural and Urban residents. The problem of the vulnerable subpopulation being excluded from the system may be based on the findings that older adults or those individuals with no schooling who are living alone with cognitive impairment were strongly associated with higher odds of catastrophic health expenditure. Thus, efforts are needed to ensure that all adults are covered by health insurance to improve the health and well-being of these vulnerable older adults.

Our findings provide new evidence to inform the development of health care policies on social health insurance and medical assistance in consideration of these vulnerable adults. Policy-makers may consider providing health protection and developing economic assistance programs targeting subpopulations, particularly for those individuals having low socioeconomic status. For example, these programs may include increasing subsidies, increasing the coverage of medical insurance for chronic diseases and physical disability, monitoring health and implementing prevention strategies for adults living alone with cognitive impairment.

The major strengths of this study included the large sample size from a national survey of middle-aged and older adults in China, which allowed us to identify a unique subpopulation. In addition, we used propensity score matching to strictly address the imbalance and confounding effects [46–48] and observed low standardized mean differences in most of the factors, thus further strengthening the findings. Furthermore, the outcomes that were considered in this study have been previously well defined and validated [6, 9].

However, there were several limitations in the study. First, although we pooled observations from different waves of the CHARLS, the analyses had natural cross-sectional features, which restricted causal conclusions from being obtained. Furthermore, due to the fact that we only focused on the CHARLS samples, our findings were not generalizable to the general Chinese population. Second, recall biases are inevitable in questionnaire-based surveys, especially for the outcomes including OOP health care costs. Third, there was potential contamination in the cases and matched comparators, as the participants’ cohabitation and cognitive function may vary across the waves.

## Conclusion

This was a population-level study focusing on adults living alone with cognitive impairment in the context of rapid population ageing and traditional cultural norms. This study demonstrated that these Chinese adults living alone with cognitive impairment in the CHARLS experienced a high burden of catastrophic health expenditure. Health care policies on social health insurance and medical assistance should consider these vulnerable older adults.

## Supplementary Information

**Additional file 1.**

## Data Availability

The datasets supporting the conclusions of this article is available in the [CHARLS] repository (http://charls.pku.edu.cn). If someone want to request data from this study, please contact professor Zuyun Liu, corresponding author of this paper.

## Abbreviations

CHARLS: China Health and Retirement Longitudinal Study
OOP: Out-of-pocket
TICS: Telephone Interview of Cognitive Status
URBMI: Urban Resident Basic Medical Insurance
OR: Odd ratio
CI: Confidence interval
SD: Standard deviation
SMD: Standardised mean difference
SE: Standard error
i.e.: Id est.
e.g.: Exempli gratia
et al.: Et alia
