# Supplementary material for: Catastrophic health expenditure among Chinese adults living alone with cognitive impairment: findings from the CHARLS
Source: BMC Geriatr. 2022 Aug 4;22:640. doi: 10.1186/s12877-022-03341-8 (PMC9351200; doi:10.1186/s12877-022-03341-8)
Supplement: Supplementary file 1 — Additional file 1. [file 12877_2022_3341_MOESM1_ESM.docx]

**Table S1. Additional analysis 1: setting the cutoff of cognitive impairment as a standard deviation below mean at every age: Catastrophic health expenditure in the cases (i.e., living alone with cognitive impairment) and the matched comparators (i.e., living with others and with normal cognition) in CHARLS 2011-2018.**

|  | **OR (95% CI)** | ***P* value** |
| --- | --- | --- |
| **Catastrophic health expenditure** | 1.34 (1.00, 1.81) | 0.053 |

Note: OR, odds ratio; CI, confidence interval.

Generalized estimating equation models were used for catastrophic health expenditure. Results were from a model adjusted for age, sex, marital status, residence areas, alcohol consumption, smoking status, education, disease counts.


**Table S2. Additional analysis 2: catastrophic health expenditure in the cases (i.e., living alone with cognitive impairment) and the matched comparators (i.e., living with others and with normal cognition) when adding ADL status as a covariate in CHARLS 2011-2018.**

|  | **Model 1**  **OR (95% CI)** | ***P* value** | **Model 2**  **OR (95% CI)** | ***P* value** |
| --- | --- | --- | --- | --- |
| **Catastrophic health expenditure** | 1.72 (1.28, 2.31) | <0.001 | 1.74 (1.29, 2.34) | <0.001 |

Note: OR, odds ratio; CI, confidence interval; ADL, activities of daily living.

Generalized estimating equation models were used for catastrophic health expenditure. Model 1 adjusted for age and sex. Model 2 additionally adjusted for marital status, residence areas, alcohol consumption, smoking status, education, and disease counts based on Model 1.

ADL status covered 6 items: defecation, using the toilet, getting in/out of bed, eating, bathing, and dressing.The answers to each of the questions (i.e., items above) were divided into 4 levels: “I can not do it”, “Yes, I have difficulty and need help”, “I have difficulty but can still do it”, and “No, I don’t have any difficulty”. ADL status was defined as a binary variable, with a value of 0 if the observations reported “No, I don’t have any difficulty” for all the items covered in ADL status and 1 if otherwise.

ADL status was included as a covariate in propensity score matching. New result of number of observations are listed below: cases (N = 575) and matched comparators (N = 892).

**Table S3. Additional analysis 3: catastrophic health expenditure in the cases (i.e., living alone with cognitive impairment) and the matched comparators (i.e., living with others and with normal cognition) without adjusting for marital status in CHARLS 2011-2018.**

|  | **Model 1**  **OR (95% CI)** | ***P* value** | **Model 2**  **OR (95% CI)** | ***P* value** |
| --- | --- | --- | --- | --- |
| **Catastrophic health expenditure** | 1.91 (1.42, 2.57) | <0.001 | 1.90 (1.41, 2.56) | <0.001 |

Note: OR, odds ratio; CI, confidence interval.

Generalized estimating equation models were used for catastrophic health expenditure. Model 1 adjusted for age and sex. Model 2 additionally adjusted for residence areas, alcohol consumption, smoking status, education, and disease counts based on Model 1.


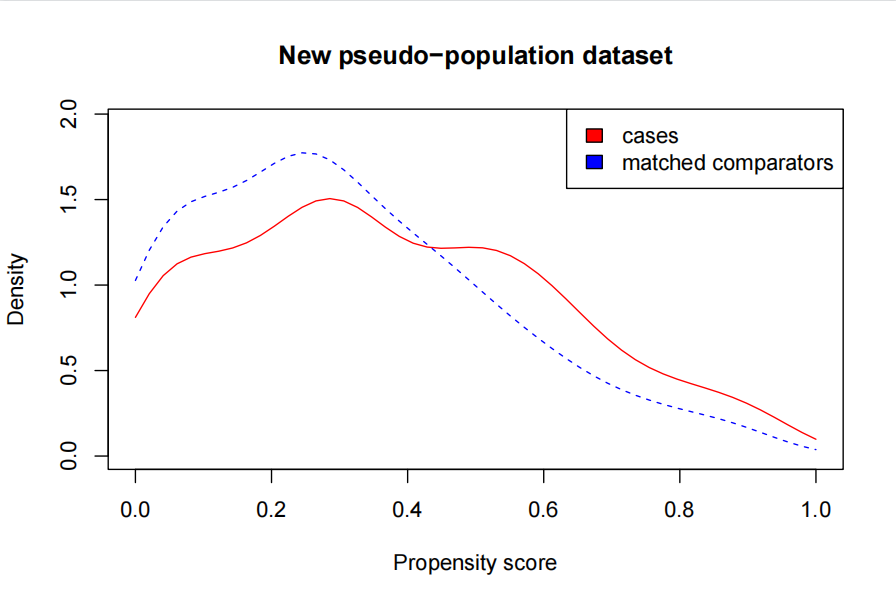


**Figure S1. Kernel density of cases (i.e., living alone with cognitive impairment) and the matched comparators (i.e., living with others and with normal cognition) to check the overlap within the new pseudo-population dataset.**
